# Growth patterns in Onychophora (velvet worms): lack of a localised posterior proliferation zone

**DOI:** 10.1186/1471-2148-10-339

**Published:** 2010-11-04

**Authors:** Georg Mayer, Chiharu Kato, Björn Quast, Rebecca H Chisholm, Kerry A Landman, Leonie M Quinn

**Affiliations:** 1 Institute of Biology II: Animal Evolution & Development, University of Leipzig, Talstrasse 33, D-04103 Leipzig, Germany; 2 Institut für Evolutionsbiologie und Ökologie, Universität Bonn, An der Immenburg 1, D-53121 Bonn, Germany; 3 Department of Mathematics and Statistics, University of Melbourne, Victoria 3010, Australia; 4 Department of Anatomy and Cell Biology, University of Melbourne, Victoria 3010, Australia

## Abstract

**Background:**

During embryonic development of segmented animals, body segments are thought to arise from the so-called "posterior growth zone" and the occurrence of this "zone" has been used to support the homology of segmentation between arthropods, annelids, and vertebrates. However, the term "posterior growth zone" is used ambiguously in the literature, mostly referring to a region of increased proliferation at the posterior end of the embryo. To determine whether such a localised posterior proliferation zone is an ancestral feature of Panarthropoda (Onychophora + Tardigrada + Arthropoda), we examined cell division patterns in embryos of Onychophora.

**Results:**

Using in vivo incorporation of the DNA replication marker BrdU (5-bromo-2'-deoxyuridine) and anti-phospho-histone H3 immunolabelling, we found that a localised posterior region of proliferating cells does not occur at any developmental stage in onychophoran embryos. This contrasts with a localised pattern of cell divisions at the posterior end of annelid embryos, which we used as a positive control. Based on our data, we present a mathematical model, which challenges the paradigm that a localised posterior proliferation zone is necessary for segment patterning in short germ developing arthropods.

**Conclusions:**

Our findings suggest that a posterior proliferation zone was absent in the last common ancestor of Onychophora and Arthropoda. By comparing our data from Onychophora with those from annelids, arthropods, and chordates, we suggest that the occurrence of a "posterior growth zone" currently cannot be used to support the homology of segmentation between these three animal groups.

## Background

The most obvious subdivision of the body into serially repeated units or segments occurs in annelids (ringed worms), panarthropods (onychophorans, tardigrades and arthropods), and chordates (including vertebrates, urochordates and cephalochordates). During embryonic development, segments are commonly believed to originate from the so-called "posterior growth zone" (review [1]). However, this term has been applied very broadly in the past, which has resulted in ambiguity. For example, the occurrence of a "posterior growth zone" has been used to support the homology of segmentation either specifically in annelids and panarthropods [2-4] or in all three groups of segmented animals, suggesting that segmentation was present in their last common ancestor [1,5-8].

Traditionally, the term "posterior growth zone" has been used to describe a localised and highly proliferative terminal body region, which has been dubbed the "proliferating area" or "zone of proliferation" [9-11]. While it seems clear that such a localised proliferation zone is present in embryos, larvae, or juveniles of annelids, including clitellates and polychaetes [11-18], the situation is less clear for chordates. In vertebrate embryos, a higher proliferative activity, as compared to the pre-somitic mesoderm region, consistent with the presence of stem cells has been observed in the tailbud [19-24]. In cephalochordate embryos, the pre-somitic mesoderm region is absent, but the tailbud shows a high number of proliferating cells during somitogenesis [25,26]. In contrast, a "posterior growth zone" is lacking completely from embryos of urochordates [1], as evidenced by various cell lineage and cell proliferation studies [27-29]. Thus, the ancestral state for the chordates remains unclear.

Apart from annelids and vertebrates, a pool of proliferating cells, or stem-like cells, at the posterior end have been proposed for the arthropod embryos [3,4,11,30]. However, the existence of such a localised zone has only been confirmed for embryos of malacostracan crustaceans [31-33]. Although the malacostracan stem-like cells are reminiscent of the clitellate teloblasts, their homology is questionable [4,31,32,34,35]. Leaving aside the issue of the homology of crustacean and clitellate teloblasts, the existence of a posterior pool of proliferating cells has been doubted for all remaining arthropod groups [35-40]. Thus, the question arises of whether a localised posterior proliferation zone is an ancestral feature of (pan)arthropods. To clarify this question, an analysis of the pattern of cell division in embryos of a closely related outgroup, such as Onychophora or velvet worms, is required.

So far, specific markers of dividing cells have not been used to investigate the mode of axis elongation in onychophoran embryos, which instead has been deduced from classical histological methods and scanning electron microscopy. Based on these studies, it is generally assumed that there is a distinct posterior proliferation zone in onychophoran embryos [10,11,41,42]. However, the original illustrations [10,43-49] do not bear this out, and the ancestral mode of cell proliferation and axis elongation in Panarthropoda has remained obscure. Despite this, others have assumed all arthropods have a restricted posterior proliferation zone. Indeed, Jaeger and Goodwin [50,51] have developed mathematical models based on the concept of a proliferation zone to investigate the dynamics of sequential addition of segments during development in segmented animals, including the arthropods.

To clarify whether a posterior proliferation zone exists in Onychophora, we analysed the cell division patterns in embryos from the two major onychophoran groups: the Peripatidae and the Peripatopsidae. Our data demonstrate the absence of a posterior proliferation zone in the last common ancestor of Onychophora and Arthropoda. We have therefore modified the mathematical segmentation model of Jaeger and Goodwin [50,51] by assuming distributed, rather than localised, cell proliferation during development.

## Results and discussion

### Anti-BrdU immunolabelling does not reveal a posterior proliferation zone in Onychophora

**Figure 1:**
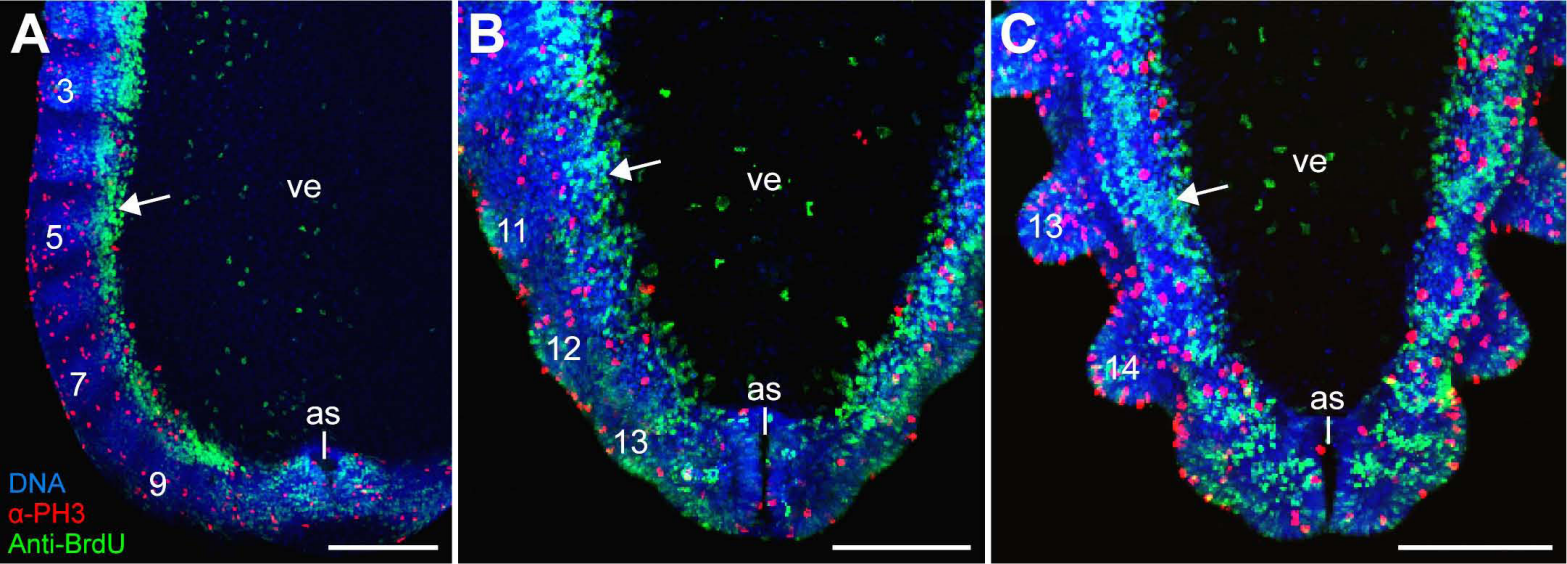
**Incorporation of 5-bromo-2'-deoxyuridine (BrdU) and subsequent immunohistochemical detection at the posterior end in embryos of the onychophoran *Euperipatoides rowelli *(Peripatopsidae)**. Triple-labelling with the DNA-selective dye Hoechst (Bisbenzimide, blue), and anti-BrdU (green) and anti-phospho-histone H3 antibodies (α-PH3, red) after 3 hours incubation in BrdU. Confocal micrographs. Arrows point to areas of intense anti-BrdU immunolabelling corresponding to the developing ventral organs. Note that neither anti-BrdU nor α-PH3 immunolabelling reveals an increased number of cell divisions at the posterior end. (A) Posterior end of an early stage 3 embryo. (B) Posterior end of a late stage 3 embryo. (C) Posterior end of a stage 4 embryo. Leg-bearing segments numbered. Abbreviations: as, anus; ve, ventral extra-embryonic ectoderm. Scale bars: A-C, 200 μm.

In vivo incorporation of the DNA replication marker BrdU, in conjunction with anti-BrdU immunolabelling, is a commonly used method for analysing embryonic cell division patterns [15,52-59]. Among annelids, anti-BrdU immunolabelling revealed a distinct posterior proliferation zone in post-metamorphic stages of polychaetes, including the echiurans [15,16,54]. A similar localised region containing stem-like cells or teloblasts also occurs in clitellate embryos [13,14,18]. To obtain comparative data from Onychophora, we applied the anti-BrdU immunolabelling in elongating embryos of the velvet worm species *Euperipatoides rowelli*. In contrast to annelids, we did not detect a higher number of BrdU labelled cells at the posterior end of the onychophoran embryos than in the rest of the body (Figures 1A-C). Thus, this method does not confirm the existence of a posterior proliferation zone in Onychophora.

### Anti-BrdU immunolabelling is not specific to dividing cells

It was possible we were unable to detect the posterior proliferation zone in onychophoran embryos as a consequence of non-specific incorporation of BrdU into actively dividing cells. However, extensive work has shown that BrdU will be incorporated into all cells undergoing DNA synthesis, including endocycling cells [60-63]. The latter are specialised cells, which increase their biosynthetic activity by entering endocycles, i.e., successive rounds of DNA replication without an intervening mitosis [63-65]. Due to ongoing DNA synthesis in these cells, BrdU is incorporated in their nuclei; although these cells can grow larger they do not divide.

**Figure 2:**
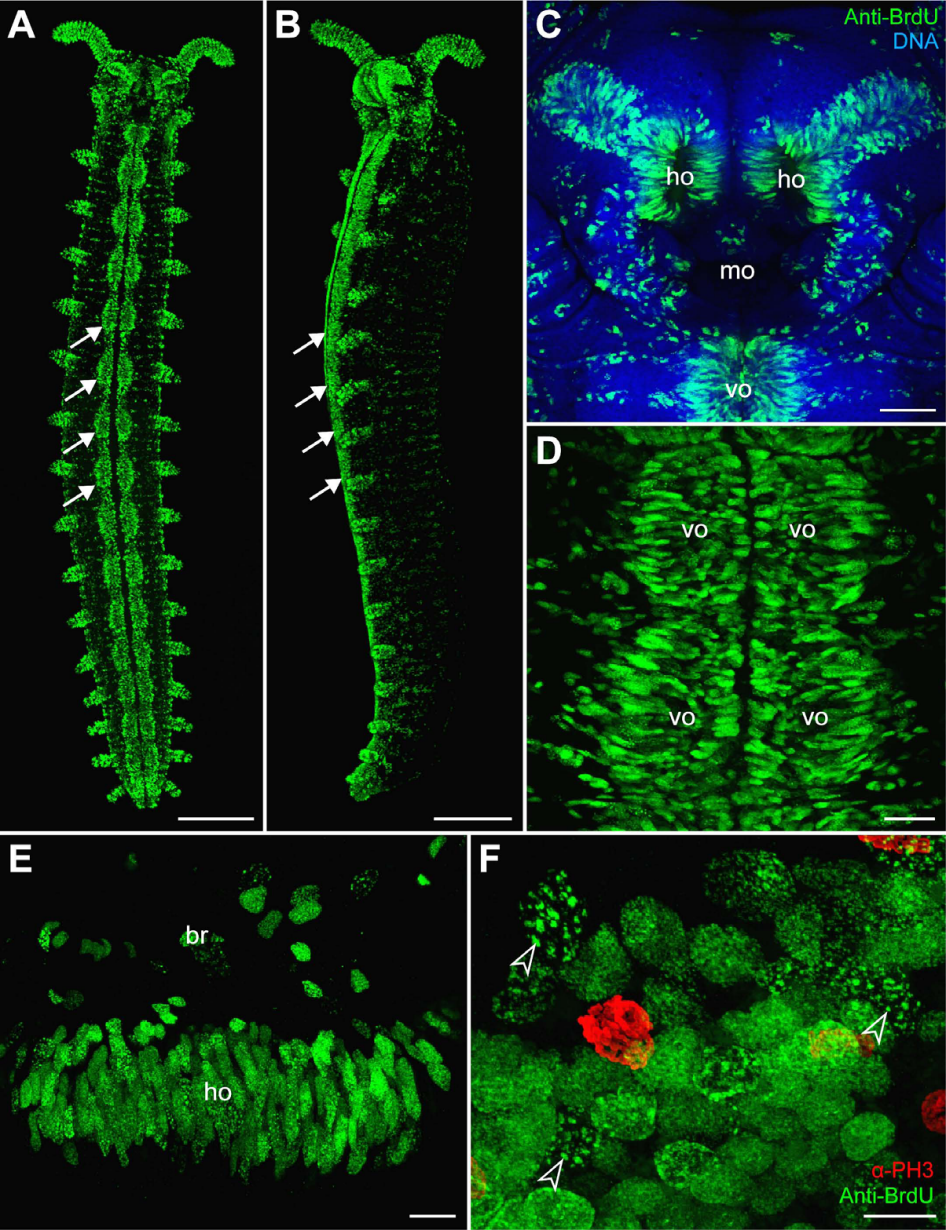
**Incorporation of 5-bromo-2'-deoxyuridine (BrdU) and subsequent immunocytochemical detection in embryos of the onychophoran *Euperipatoides rowelli *(Peripatopsidae)**. Confocal micrographs. Note the intense labelling in the ventral organs and anlagen of the hypocerebral organs, which are derivatives of the anterior-most pair of ventral organs. (A, B) Ventral and lateral views of a late stage 6 embryo after 12 hours incubation in BrdU. Arrows point to the ventral organs. (C) Ventral view of the head of a late stage 6 embryo after 10 hours incubation in BrdU. Double-labelling with the DNA-selective dye Hoechst (Bisbenzimide, blue) and anti-BrdU antibody (green). (D) Detail of two pairs of ventral organs from a stage 6 embryo after 12 hours incubation in BrdU. (E) Optical cross-section of an anlage of the hypocerebral organ after 12 hours incubation in BrdU showing a superficial layer of anti-BrdU labelled cells. (F) Detail of ventral organ nuclei after 3 hours incubation in BrdU (ventral view). Double-labelling with anti-BrdU (green) and anti-phospho-histone H3 antibodies (α-PH3, red). Arrowheads point to BrdU incorporation foci in each nucleus. Abbreviations: br, presumptive brain tissue; ho, anlagen of hypocerebral organs; mo, presumptive mouth opening; vo, ventral organs. Scale bars: A and B, 500 μm; C, 100 μm; D, 50 μm; E and F, 20 μm.

Our BrdU-labelling experiments on onychophoran embryos revealed specific labelling patterns corresponding to some developing structures and organs (Figures 2A-E). In particular, the so-called ventral organs and the anlagen of their derivatives, the hypocerebral organs, show a high number of BrdU-positive cells, with virtually every cell labelled in the superficial layer (Figures 2A-E). The nuclei of the BrdU-positive cells show a divergent morphology compared to other cells since they are columnar in shape, larger in size, and contain conspicuously condensed chromatin [66,67]. Notably, increased size and condensed chromatin is a feature of other endoreplicating cells, e.g., the salivary gland cells and nurse cells in *Drosophila melanogaster *[68,69]. Furthermore, our data show that in the ventral organs BrdU is initially incorporated in a conspicuous punctate pattern (Figure 2F), which is typical of endocycling cells [62]. Due to this peculiar pattern of BrdU incorporation and modified cell morphology, we suggest that most cells in the ventral organs (and in the hypocerebral organ anlagen) are endocycling cells. Since we cannot exclude the possibility that other embryonic cells also enter the endocycle, we caution that anti-BrdU immunolabelling will not provide a definitive method for detecting mitotic cell division patterns in onychophoran embryos.

### Absence of a posterior proliferation zone in Onychophora

**Figure 3:**
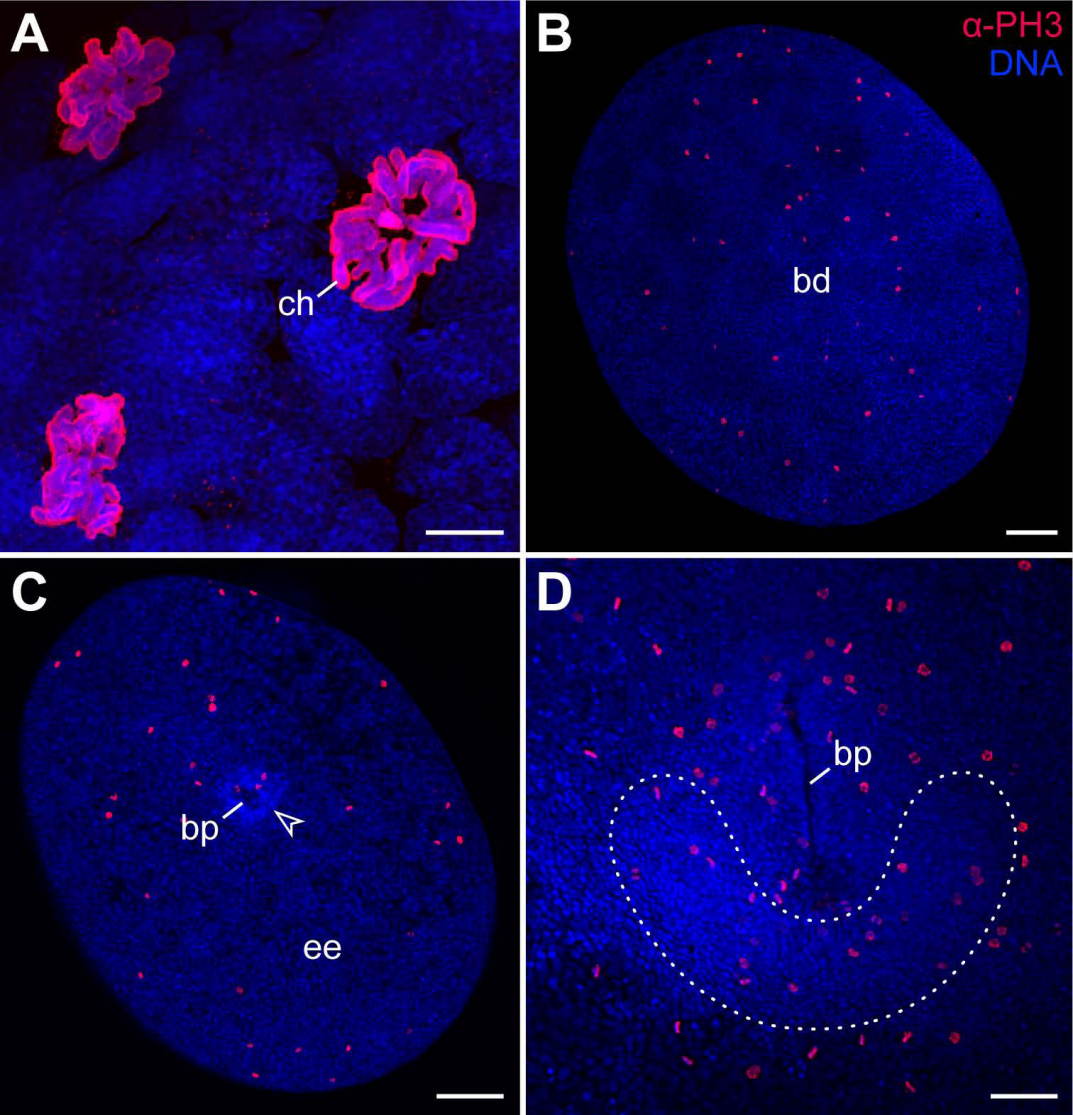
**Cell division patterns in early embryos of the ovoviviparous onychophoran *Euperipatoides rowelli *(Peripatopsidae)**. Double-labelling with the DNA-selective dye Hoechst (Bisbenzimide, blue) and anti-phospho-histone H3 antibody (α-PH3, red). (A) Mitotic cells from an early segmenting embryo. The nuclei of Hoechst stained cells show diffuse chromatin whereas chromosomes (ch) are seen in Hoechst/α-PH3 double-labelled cells. (B) Blastula stage embryo with mitotic cells scattered throughout the blastoderm (bd). (C) Early gastrula stage embryo with dividing cells in the extra-embryonic ectoderm (ee) and in the germ disc (arrowhead). (D) Early germ band embryo with cell divisions scattered throughout the germ disc. Paired germ band (dotted line) extends anteriorly on each side of the slit-like blastopore (bp). Abbreviations: bd, blastoderm; bp, blastopore; ch, chromosomes; ee, extra-embryonic ectoderm. Scale bars: A, 10 μm; B and C, 200 μm; D, 100 μm.

As the BrdU-labelling experiments revealed a large number of non-dividing, endoreplicating cells and cell lineage analyses are not applicable to onychophoran embryos, we next used an anti-phospho-histone H3 (α-PH3) antibody to determine whether there is a concentration of mitotic cell divisions in the posterior of the onychophoran embryo. This antibody specifically recognises mitotic figures in prophase, metaphase and anaphase [70] and, thus, allows detection of mitotic cells in various animals, including the onychophorans (Figure 3A) [66]. To obtain a comprehensive picture of cell division patterns during development, we analysed numerous embryos (n = 187) of the onychophoran *Euperipatoides rowelli *(Peripatopsidae) at consecutive developmental stages and covering all embryonic stages [42].

**Figure 4:**
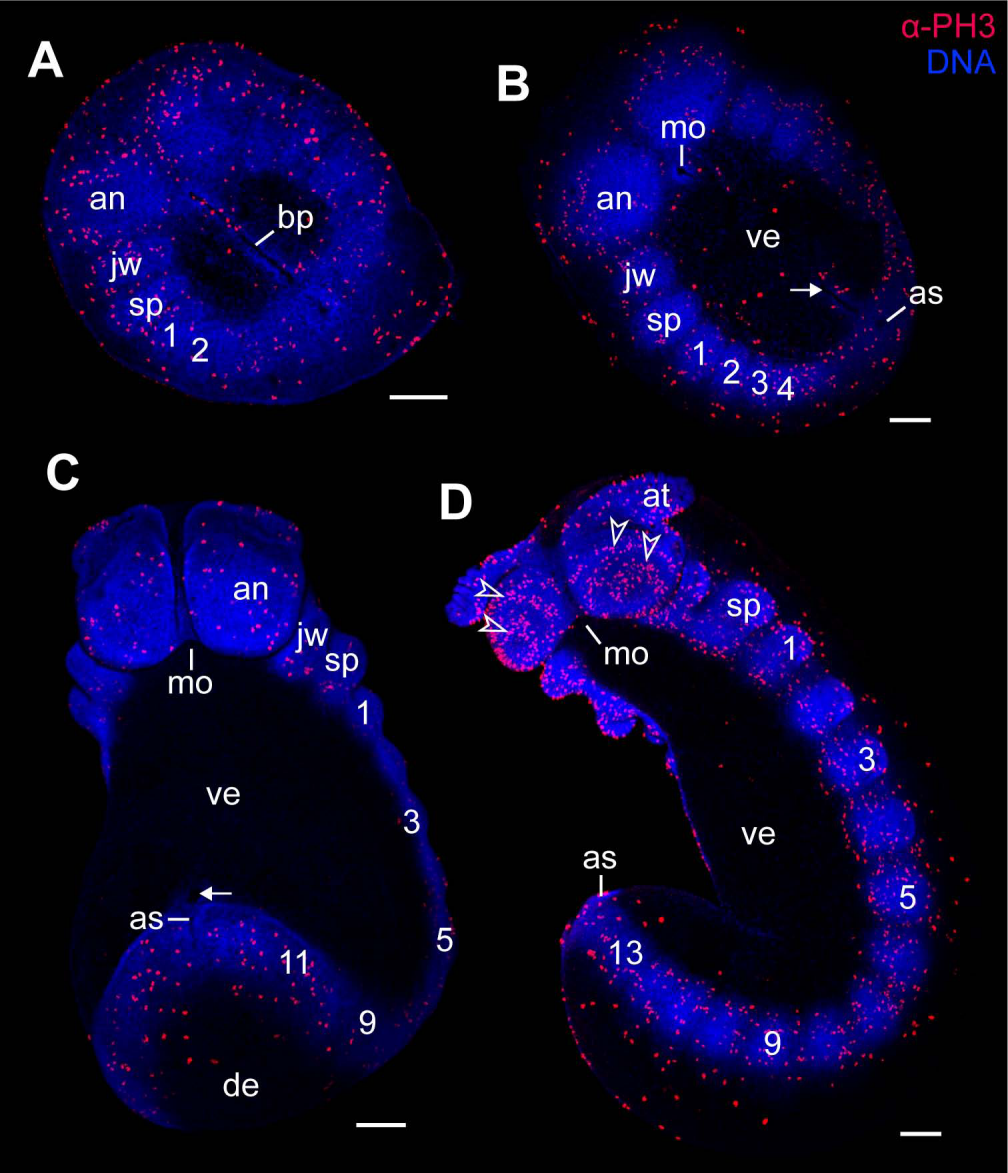
**Cell division patterns in segmenting embryos of the ovoviviparous onychophoran *Euperipatoides rowelli *(Peripatopsidae)**. The full number of 15 leg-bearing segments has not been established yet. Double-labelling with the DNA-selective dye Hoechst (Bisbenzimide, blue) and anti-phospho-histone H3 antibody (α-PH3, red). (A) Flat preparation of an embryo with two leg-bearing segments. (B) Flat preparation of an embryo with four leg-bearing segments. (C) Ventrolateral view of a late stage 3 embryo with 11 leg-bearing segments. (D) Ventrolateral view of an early stage 4 embryo with 13 leg-bearing segments. Note the concentric rings of proliferating cells in the antennal segment (arrowheads). Arrows (in B and C) point to remnants of the blastopore in front of the future anus. Leg-bearing segments numbered. Abbreviations: an, antennal segment; as, anus; at, presumptive antenna; bp, blastopore; de, dorsal extra-embryonic ectoderm; jw, jaw segment/presumptive jaw; mo, embryonic mouth; sp, slime papilla segment/presumptive slime papilla; ve, ventral extra-embryonic ectoderm. Scale bars: A-D, 200 μm.

Our data show that the mitotic cells are scattered throughout the blastoderm at the blastula stage (Figure 3B). This apparently random distribution of dividing cells persists until the blastopore arises in the newly formed germ disc at the gastrula stage (Figure 3C). From this stage onwards, the number of dividing cells increases within the entire germ disc, but we do not see a concentration of dividing cells at the posterior end of the embryo (Figure 3D). Even when the embryo continues to elongate during development, the number of dividing cells does not become higher at the posterior end (Figures 1A-C and 4A-D), even though new segments are segregated in this body region [42] (see also [71] for the expression pattern of *engrailed *and *wingless *in embryos of a closely related species, *Euperipatoides kanangrensis*). The data therefore suggest that the posterior end of *Euperipatoides rowelli *embryos does not contain a zone of a higher proliferative activity.

To clarify whether the absence of a concentrated posterior proliferation zone is a common feature of Onychophora, we studied embryogenesis in *Epiperipatus isthmicola*, a representative of Peripatidae. Our data show that the cell division pattern in embryos of *Epiperipatus isthmicola *(n = 124) is similar to that in *Euperipatoides rowelli*, with dividing cells scattered along the body throughout development (Figues 5A-E). Thus, our results from two distantly related species of Onychophora suggest that, in contrast to previous assumptions [3,4,10,11,72,73], a distinct concentration of dividing cells, which would denote a posterior proliferation zone, does not exist in this group.

**Figure 5:**
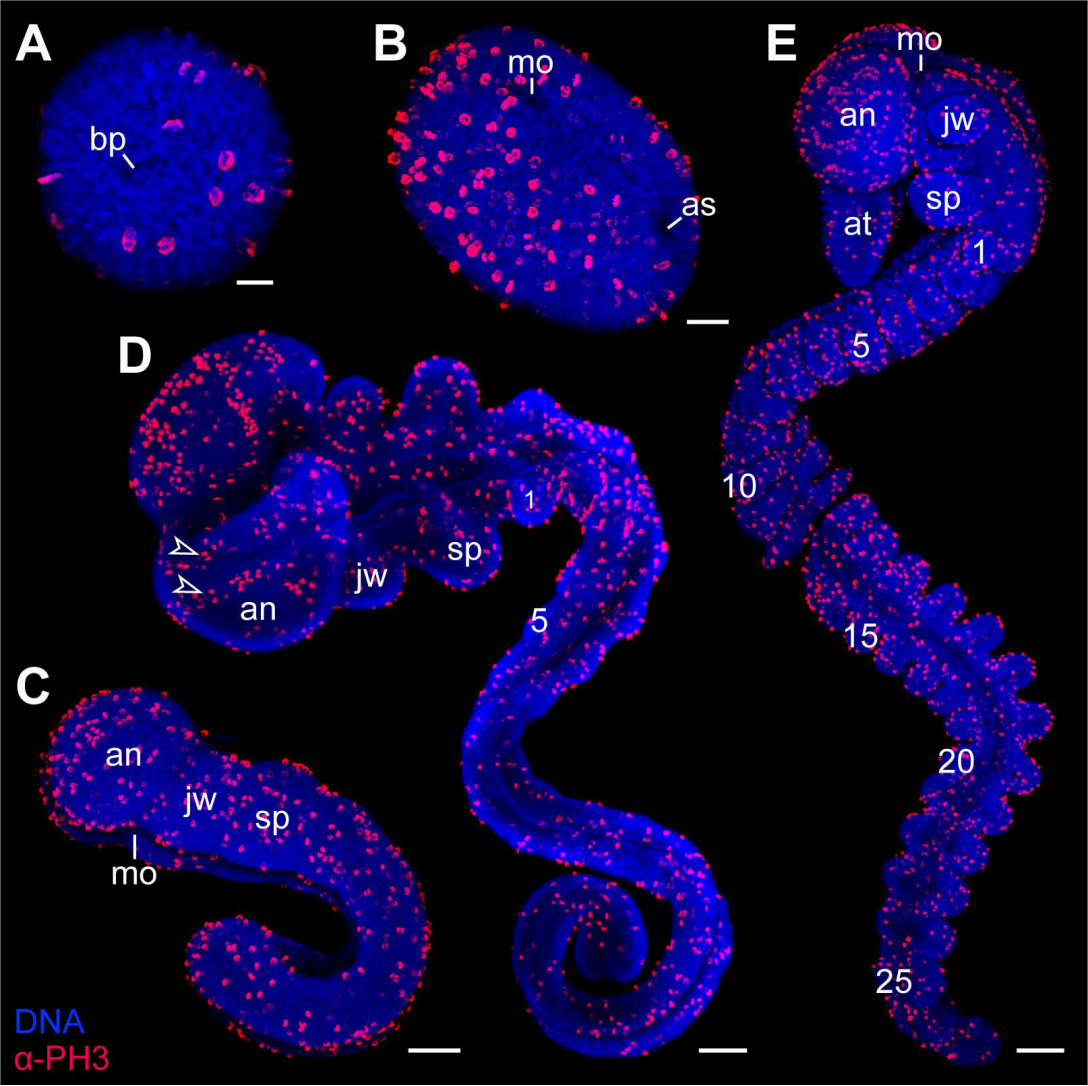
**Cell division patterns in embryos of the placental viviparous onychophoran *Epiperipatus isthmicola *(Peripatidae)**. The full number of leg-bearing segments (30-32 in females and 27-29 in males) has not been established yet. Double-labelling with the DNA-selective dye Hoechst (Bisbenzimide, blue) and anti-phospho-histone H3 antibody (α-PH3, red). (A) Gastrula stage embryo. (B) Elongating germ band embryo with separate mouth and anus openings. (C) Elongating embryo with 10 leg-bearing segments. (D) Elongating "coiled stage" embryo with 20 leg-bearing segments. Arrowheads indicate concentric rings of proliferating cells in the antennal segment. (E) A "coiled stage" embryo with 26 leg-bearing segments. Abbreviations: an, antennal segment; as, anus; at, presumptive antenna; bp, blastopore; jw, jaw segment/presumptive jaw; mo, embryonic mouth; sp, slime papilla segment/presumptive slime papilla. Scale bars: A, 25 μm; B, 50 μm; C and D, 100 μm, E, 150 μm.

### No posterior proliferation zone in the last common ancestor of Panarthropoda

**Figure 6:**
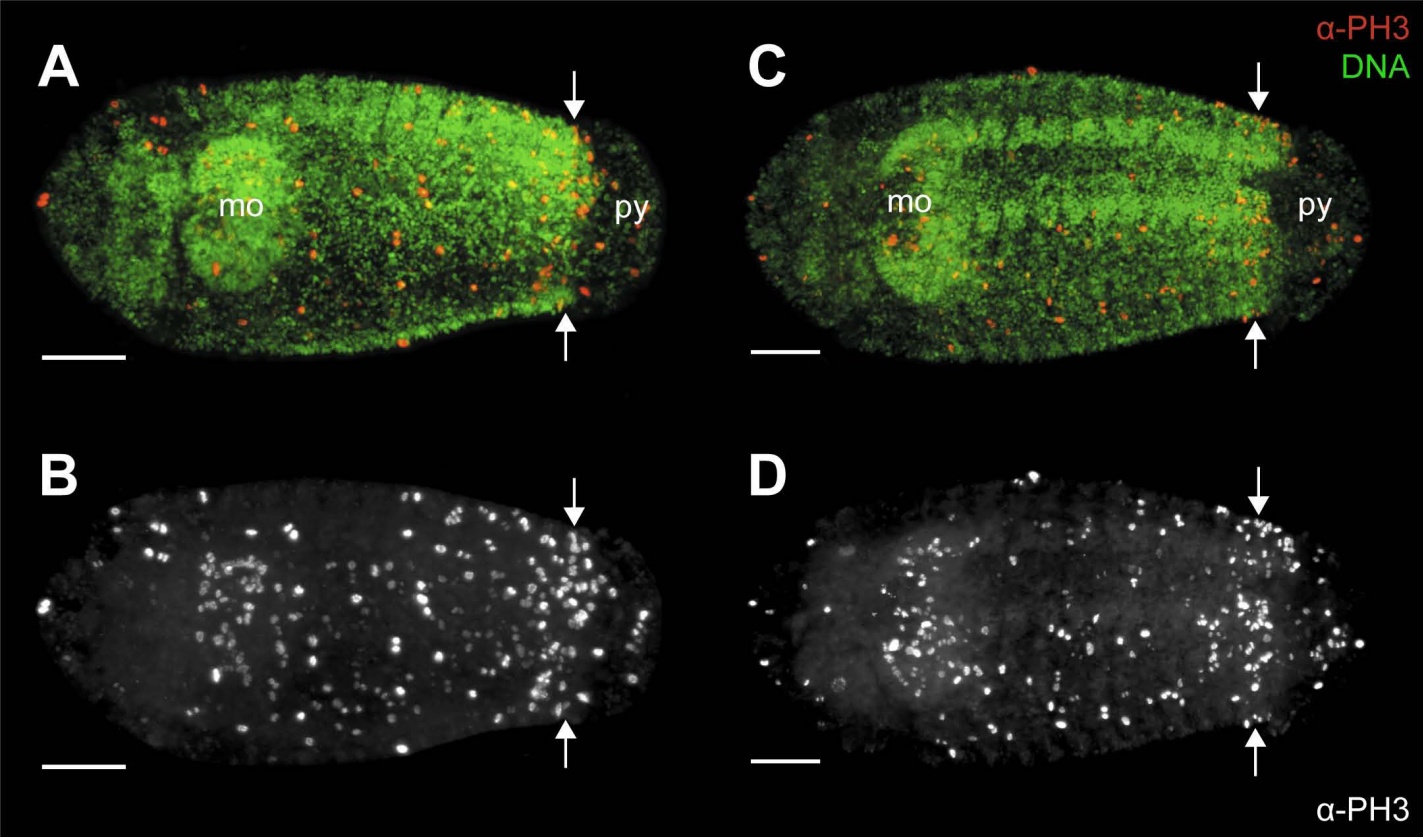
**Cell division pattern in larvae of the polychaetous annelid *Capitella teleta *(Scolecida, Capitellidae)**. Confocal maximum projections of stage 8 larvae in ventral (A and B) and ventrolateral views (C and D); anterior is left. Double-labelling with an α-PH3 antibody (red) and DNA-selective dye (green). Note a localised region of high cell proliferation (arrows) in front of the pygidium (py). Abbreviations: mo, presumptive mouth; py, pygidium. Scale bars: 50 μm.

**Figure 7:**
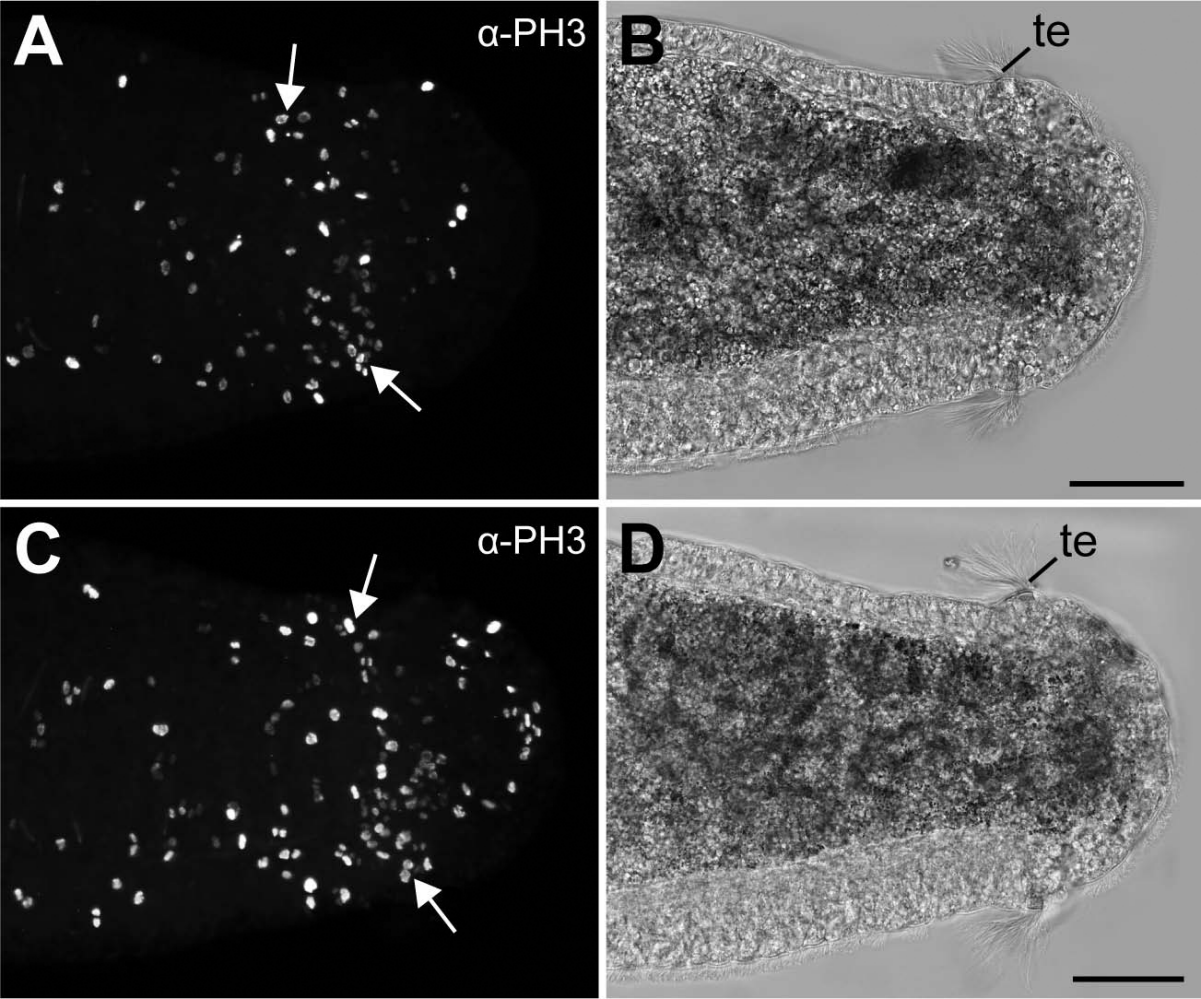
**Cell division pattern at the posterior end in larvae of the polychaetous annelid *Capitella teleta *(Scolecida, Capitellidae)**. Confocal (A, C) and light micrographs (B, D) of posterior ends of an early (A, B) and a late stage 8 larvae (C, D), labelled with an α-PH3 antibody. Note a localised region of high cell proliferation (arrows) in front of the telotroch (te). Scale bars: 50 μm.

In summary, the results of our study show that mitotic figures do not occur in a condensed pattern at the posterior end in onychophoran embryos, suggesting that there is no preferential zone of proliferation in this body region. It is unlikely that the α-PH3 immunolabelling method we used failed to detect the posterior proliferation zone since this technique reliably shows such a zone in larvae of the annelid *Capitella teleta *(Figures 6A-D and 7A-D), as does the anti-BrdU immunolabelling in late larval stages and juveniles of the same species [15]. Furthermore, the α-PH3 immunolabelling method revealed other proliferating regions rather than a posterior proliferation zone in onychophoran embryos. For example, concentric rings of proliferating cells, which correspond in timing and position with the anlagen of the hypocerebral organs [66], are found in the antennal segment of the onychophoran embryos (Figures 4D and 5D).

The lack of evidence for a localised posterior proliferation zone in Onychophora corresponds well with the apparent absence of such a zone in tardigrades [74,75] and most arthropods [35-40], excepting the malacostracan crustaceans. We therefore suggest that a localised posterior proliferation zone was absent in the last common ancestor of Panarthropoda.

### Modified mathematical model suggests that a posterior proliferation zone is not required for segmentation

One of the assumptions of Jaeger and Goodwin's [50,51] segmentation model is that cell proliferation occurs only at the posterior end of the segmenting embryo. However, the results of our and other studies [35,38,75] revealed that a higher concentration of mitotic cells does not occur at the posterior end in embryos of onychophorans, tardigrades and most arthropods. We therefore modified the Jaeger and Goodwin mathematical model [50,51] and assume distributed proliferation of cells along the embryo. We retain an anterior-to-posterior developmental gradient in our model as it occurs in embryos of short germ developing arthropods and onychophorans, which contrasts with the situation found in long germ developing insects, in which all segments arise simultaneously [38]. As indicated by our experimental data, we choose a uniform (constant) proliferation rate across the entire embryo.

**Figure 8:**
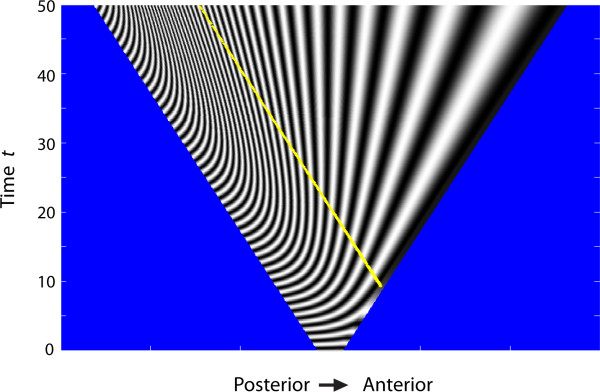
**Space time diagram of cell state as a function of position (horizontal axis) and time (vertical axis) in a uniformly proliferating tissue with a linear growth rate**. Due to proliferation throughout the tissue, the length increases with time *t *as *L(t) = t *+ 2. The parameters in the Jaeger and Goodwin model [50,51] are set as *A *= 0.5, *B *= 5, *T_0 _*= 1 for 0 <*t *< 50. The cell state ranges from -10 <*z *< 10 and this is represented on the graph as shading between black and white (corresponding to *z *= -10, *z *= 10 respectively). The region above the dotted yellow line has segmented. Areas outside the tissue are coloured in blue.

At early times, all cells across the developing tissue oscillate between states (Figure 8). After some time, however, the anteriorly located cells in the tissue begin to increase the length of their oscillation period, and the first segment establishes in the anterior-most region just before *t *= 10. Subsequently, additional segments are established adjacent to preceding segments in an anterior-to-posterior progression. Moreover, there is a decrease in size of the newly established segments from anterior to posterior end. Here, since the cells within a segment continue to proliferate, the established segments also grow in width. This finding corresponds well with the observed external and internal anatomy of the embryos studied (Figures 4A, B and 5D).

Taken together, the results of our mathematical model show that segments can be patterned successfully without the involvement of a localised posterior proliferation zone in embryos of short germ developing arthropods and onychophorans.

## Conclusions

The term "posterior growth zone" is commonly used to describe the terminal body region, which gives rise to segments in embryos of most panarthropods, annelids, and chordates [1,4,5,7,11,76]. However, according to our results, the "posterior growth zone" of panarthropods is not a localised "zone" of proliferation but rather an area, in which segments are patterned, as evidenced by various gene expression data available from various arthropods [35,77-81]. This contrasts with the "posterior growth zone" of annelids, in which both an increased number of cell divisions and segment patterning occur [13-18,82]. With respect to vertebrates, the term "posterior growth zone" is applied in different ways and refers either to the tailbud, which proliferates cells for somites, or to the pre-somitic mesoderm area, which establishes segmental borders [1,22,24,83-85]. Since the terminal body region differs considerably in composition and extent among panarthropods, annelids, and chordates, the term "posterior growth zone" is imprecise and therefore cannot be used to support the homology hypothesis [1] of segmentation between these three animal groups.

## Materials and methods

### Specimens and embryos

Females of the onychophoran species *Euperipatoides rowelli *Reid, 1996 and *Epiperipatus isthmicola *(Bouvier, 1905) were collected and maintained in the laboratory as described previously [67,86]. Females were dissected at various times of the year to obtain a range of consecutive developmental stages. Embryos were staged according to previous descriptions of onychophoran embryogenesis [42,66,67,87,88]. For positive controls, *Capitella teleta *Blake, Grassle & Eckelbarger 2009 ("*Capitella *sp. I" *sensu *[15]) larvae and juveniles were obtained from a culture at the Department of Evolutionary Biology (University of Bonn, Germany). The animals were reared in 20 × 20 cm plastic boxes containing 1 cm sieved mud (500 μm) covered with 4 cm ultrafiltrated seawater from the northern Wadden Sea at 18°C. Water and sediment were changed every two weeks and the boxes aerated continuously. To obtain developmental stages, brood tubes were taken from the sediment and opened with minute needles.

### Anti-phospho-histone H3 immunolabelling and DNA staining

Onychophoran embryos were handled as described previously [66,67]. Annelid larvae were staged according to Seaver et al. [15]. Embryos and larvae of all species studied were fixed overnight in 4% paraformaldehyde in phosphate-buffered saline (PBS; 0.1 M, pH 7.4) at 4°C and then washed in several changes of PBS and either further processed immediately or preserved in PBS containing 0.05% sodium azide for several weeks at 4°C. Pre-incubation was carried out in PBS-TX (1% bovine serum albumin, 0.05% sodium azide, and 0.5% Triton X-100 in PBS) for 1-3 hours at room temperature. Incubations with primary antibody (α-PH3; rabbit polyclonal anti-phospho-histone H3 mitosis marker; catalogue no. 06-570, Upstate, Temecula, CA, USA) and secondary antibody (goat anti-rabbit IgG conjugated to Alexa Fluorochrome 594, catalogue no. A11037, Invitrogen, Carlsbad, CA, USA) were carried out as described previously [66]. The DNA-selective fluorescent dye Hoechst (Bisbenzimide, H33258, catalogue no. 861405, Sigma-Aldrich; 1 mg/ml in PBS) was applied for 15 minutes. After several washes in PBS, the embryos and larvae were mounted in Vectashield Mounting Medium (catalogue no. H-1000, Vector Laboratories Inc., Burlingame, CA, USA) and analysed with a confocal microscope.

### Anti-BrdU and anti-phospho-histone H3 immunolabelling

To reveal DNA synthesis, the incorporation of 5-bromo-2'-deoxyuridine (BrdU; Sigma-Aldrich, St. Louis, MO, USA) was used. Onychophoran embryos were dissected and incubated for 20 minutes to 24 hours in a 0.1 mg/ml solution of BrdU (Sigma-Aldrich, St. Louis, MO, USA) in physiological saline [89] at 18°C. At the end of the incubation period, the embryos were rinsed in physiological saline and fixed for 30 minutes in 4% paraformaldehyde. DNA was denatured with a 2N HCl solution in PBS-TX for 30 minutes at room temperature. After two washes in PBS-TX, the embryos were incubated in 10% normal goat serum (Sigma-Aldrich, St. Louis, MO, USA) for 1 hour at room temperature, followed by an overnight incubation with two primary antibodies in PBS-TX at 4°C: (1) anti-BrdU monoclonal antibody (Becton Dickinson, Franklin Lakes, NJ, USA; diluted 1:50), and (2) α-PH3 antibody (as described above). After several PBS-TX washes, the embryos were incubated with two secondary antibodies (Invitrogen, Carlsbad, CA, USA), each diluted 1:500 in PBS: (1) goat anti-mouse IgG (H+L), conjugated to Alexa Fluorochrome 488 (catalogue no. A11017), and (2) goat anti-rabbit IgG conjugated to Alexa Fluorochrome 594 (catalogue no. A11037). Hoechst staining was applied as described above. After several washes in PBS, the embryos were mounted in Vectashield Mounting Medium and analysed with a confocal microscope. For controls, the embryos were treated in the same way, but without the addition of BrdU to the physiological saline. This resulted in a complete lack of anti-BrdU labelling in the nuclei. The specificity of the secondary antibody was tested by abolishing the primary antibody from the experimental procedures, which resulted in a complete lack of a positive signal within the cells. The only structures showing autofluorescence in the green and UV channels were the sclerotised claws and jaws.

### Microscopy and image processing

Embryos and larvae were analysed with the confocal laser-scanning microscopes LSM 510 META (Carl Zeiss MicroImaging GmbH) and TCS SPE (Leica Microsystems Wetzlar). The image stacks were merged digitally into partial and maximum projections with the Zeiss LSM Image Browser software (version 4.0.0.241) and ImageJ (version 1.43q). Image intensity histograms were adjusted by using Adobe (San Jose, Ca) Photoshop CS2. The adjustment was kept at a minimum to allow the micrographs of the same plate to have similar intensity. Final panels were designed with Adobe Illustrator CS2.

### Mathematical modelling

Our model adapts the one used by Jaeger and Goodwin [50,51] for animal segmentation and is based on cellular oscillators, where the phase determines the state of the cell and cells oscillate between two states. Jaeger and Goodwin [50,51] assume that there is a localised posterior proliferation zone-they call it a progress zone. In the Jaeger and Goodwin model, cells in the progress zone are oscillating in phase with each other. However, when they leave the progress zone, their oscillations slow down with their physiological age. Accordingly, the cells towards the anterior oscillate slower since they have a higher physiological age than the posterior ones. Segmentation occurs, when the state of the cell no longer oscillates and remains constant. This mechanism results in a gradient of slowing cellular oscillations and sets up a "wave" of cell state stabilisation moving in an anterior-to-posterior direction, which leads to a spatially periodic pattern of cell state that can be interpreted as sequentially forming segments.

In contrast to the Jaeger and Goodwin model [50,51], we assume that all cells have the ability to proliferate at some rate *r(x, t*), which can be a function of spatial position *x*, and time *t*. In our model, a system of discrete time equations describes the phase and period of the oscillators. It is convenient to convert the discrete time system of equations to a continuous time system, which is solved using MATLAB software (MathWorks™). We model the developing tissue in one spatial dimension, *x*, growing in time *t*. In our distributed growth model, cells can proliferate anywhere in the developing tissue. Thus, the older cells are no longer located towards the anterior of the tissues and the younger ones are no longer located towards the posterior. Accordingly, the assumed mechanism that cell oscillations slow with age [50,51] cannot result in the formation of segments in our model. We therefore modified the equations of the previous model and choose the oscillation period to be correlated with distance from the posterior end, rather than the cell age. Such positional information can be obtained from a gradient of signalling molecules in the cell's local environment. The modified cellular oscillator model gives rise to a gradient of oscillation period along the tissue length, with the faster oscillations at the posterior end.

## Competing interests

The authors declare that they have no competing interests.

## Authors' contributions

GM conceived, designed and performed the experiments on onychophorans and wrote the first draft of the manuscript. CK and BQ carried out the experiments on the *Capitella teleta *larvae and juveniles. RHC and KAL performed the mathematical modelling. LMQ provided continuous input and knowledge on cell proliferation and endoreplication. All authors participated in the discussion of the results and the preparation of the final manuscript.
